# Supplementary material for: Comparative transcriptome analyses of a late-maturing mandarin mutant and its original cultivar reveals gene expression profiling associated with citrus fruit maturation
Source: PeerJ. 2017 May 18;5:e3343. doi: 10.7717/peerj.3343 (PMC5438589; doi:10.7717/peerj.3343)
Supplement: File S1 [file peerj-05-3343-s001.zip › Supplementary File/All Supplementary Figures.doc]

A

B

C

**Fig. S1** Materials used for RNA-Seq

A, WZSTJ on the 275^th^ DAF; B, HWWZSTJ on the 275^th^ DAF; C, HWWZSTJ on the 320^th^ DAF

T1

T2

T3

**Fig. S2** Statistics of assembly length for transcripts

T1 vs T3

**A**

T2 vs T1

T2 vs T3

**B**

**Fig. S3** GO and COG classification of transcripts

A, GO categories of transcripts; B, COG classification of transcripts

**Fig. S4** A comparison between the transcripts per kilobase million from the RNA-Seq data and qPCR results

A, *CAO1*; B, *CHLM*; C, *CLH*; D, *NCED1*; E, *NCED2*; F, *PDS1*; G, *PDS2*; H, *PDS4*; I, *PSY2*;

J, *PSY5*; K, *PSY6*; L, *PSY7*. The RNA-seq log2 of expression ratio (y-axis)
have been plotted against developmental stages (x-axis).

**Fig. S5** Alignments of cDNA sequences of the *CLH* gene from HWWZSTJ and WZSTJ

W, HWWZSTJ; P, WZSTJ

**Fig. S6** Alignments of cDNA sequences of the *PSY3* gene from HWWZSTJ and WZSTJ

W, HWWZSTJ; P, WZSTJ

**Fig. S7** Alignments of cDNA sequences of the *PSY5* gene from HWWZSTJ and WZSTJ

W, HWWZSTJ; P, WZSTJ

**Fig. S8** Alignments of cDNA sequences of the *DVR* from HWWZSTJ and WZSTJ

W, HWWZSTJ; P, WZSTJ

**Fig. S9** Alignments of cDNA sequences of the *CCS* from HWWZSTJ and WZSTJ

W, HWWZSTJ; P, WZSTJ

**Fig. S10** Alignments of cDNA sequences of the *PSY6* from HWWZSTJ and WZSTJ

W, HWWZSTJ; P, WZSTJ

**Fig. S11** Alignments of cDNA sequences of the *PSY7* from HWWZSTJ and WZSTJ

W, HWWZSTJ; P, WZSTJ

**Fig. S12** Alignments of cDNA sequences of the *AB* from HWWZSTJ and WZSTJ

W, HWWZSTJ; P, WZSTJ

**Fig. S13** Phylogenetic analysis of the amino acid sequences of DVR

The tree was constructed using the Neighbor-Joining (NJ) method based on the amino acid sequences of DVR members from *Citrus sinensis* (Cs), *Morus notabilis* (Mn), *Populus trichocarpa* (Pt), *Noccaea caerulescens* (Nc), *Hevea brasiliensis* (Hb), *Glycine max* (Gm), *Arabidopsis thaliana* (At), *Lotus japonicus* (Lj), *Medicago truncatula* (Mt), *Camellia sinensis* (Ca), *Nicotiana benthamiana* (Nb), *Nicotiana tabacum* (Nt), *Solanum habrochaites* (Sh), *Sesamum indicum* (Si), *Dorcoceras hygrometricum* (Dh), *Prunus persica* (Pp), *Olea europaea* (Oe), *Mesembryanthemum crystallinum* (Mc) and *Posidonia oceanica* (Po). The DVR proteins of the WZSTJ (DVR-P) and HWWZSTJ (DVR-W) were marked with black dots. Numbers at each branch indicate the percentage support for the node among 1,000 bootstrap replicates.

**Fig. S14** Phylogenetic analysis of the amino acid sequences of CLH

The tree was constructed using the Neighbor-Joining (NJ) method based on the amino acid sequences of CLH members from *Citrus limon* (Cl), *Citrus sinensis* (Cs), *Pachira macrocarpa* (Pm), *Cajanus cajan* (Cc), *Pyrus*×*bretschneideri* (Px), *Arabidopsis thaliana* (At), *Glycine max* (Gm), *Glycine soja* (Gs), *Piper betle* (Pb), *Picrorhiza kurrooa* (Pk), *Medicago truncatula* (Mt) and *Ananas comosus* (Ac). The CLH proteins of the WZSTJ (CLH-P) and HWWZSTJ (CLH-W) were marked with black dots. Numbers at each branch indicate the percentage support for the node among 1,000 bootstrap replicates.

**Fig. S15** Phylogenetic analysis of the amino acid sequences of PSY

The tree was constructed using the Neighbor-Joining (NJ) method based on the amino acid sequences of PSY members from *Citrus sinensis* (Cs), *Citrus maxima* (Cm), *Mangifera indica* (Mi), *Manihot esculenta* (Me), *Carica papaya* (Cp), *Rhododendron molle* (Rm), *Camellia sinensis* (Cs), *Bixa orellana* (Bo), *Theobroma cacao* (Tc), *Actinidia deliciosa* (Ad), *Citrus clementina* (Cc), *Ipomoea batatas* (Ib), *Diospyros kaki* (Dk), *Citrullus lanatus* (Cl), *Dorcoceras hygrometricum* (Dh), *Prunus mume* (Pm), *Nicotiana tabacum* (Nt), *Lycium barbarum* (Lb), *Pogostemon cablin* (Pc), *Chrysanthemum boreale* (Cb), *Nicotiana langsdorffii* (Ni), *Osmanthus fragrans* (Of), *Lycium ruthenicum* (Lr), *Erythranthe lewisii* (El), *Coffea canephora* (Cc), *Momordica cochinchinensis* (Mc), *Rosa rugosa* (Rr), *Ipomoea sp. Kenyan* (Ls), *Glycine soja* (Gs), *Vitis vinifera* (Vv), *Gardenia jasminoides* (Gj), *Elaeagnus umbellata* (Eu) and *Glycine soja* (Gs).

The PSY proteins of the WZSTJ (PSY3-P, PSY5-P, PSY6-P, PSY7-P) and HWWZSTJ (PSY3-W, PSY5-W, PSY6-W, PSY7-W) were marked with black dots. Numbers at each branch indicate the percentage support for the node among 1,000 bootstrap replicates.

**Fig. S16** Phylogenetic analysis of the amino acid sequences of *NCED*

The tree was constructed using the Neighbor-Joining (NJ) method based on the amino acid sequences of NCED members from *Citrus reshni* (Cr), *Citrus sinensis* (Cs), *Citrus limon* (Cl), *Citrus unshiu* (Cu), *Theobroma cacao* (Tc), *Populus trichocarpa* (Pt), *Gossypium hirsutum* (Gh), *Vitis vinifera* (Vv), *Oxytropis ochrocephala* (Oo), *Solanum tuberosum* (St), *Solanum lycopersicum* (Sl), *Nicotiana tabacum* (Nt), *Lycium barbarum* (Lb), *Paeonia ostii* (Po), *Caragana korshinskii* (Ck), *Stylosanthes guianensis* (Sg), *Scutellaria baicalensis* (Sb), *Solanum peruvianum* (Sp), *Solanum chilense* (Sc), *Solanum ochranthum* (So) and *Lycium ruthenicum* (Lr). The NCED proteins of the WZSTJ (NCED-P) and HWWZSTJ (NCED-W) were marked with black dots. Numbers at each branch indicate the percentage support for the node among 1,000 bootstrap replicates.
