# Supplementary material for: Comparative transcriptome analyses of a late-maturing mandarin mutant and its original cultivar reveals gene expression profiling associated with citrus fruit maturation
Source: PeerJ. 2017 May 18;5:e3343. doi: 10.7717/peerj.3343 (PMC5438589; doi:10.7717/peerj.3343)
Supplement: File S1 [file peerj-05-3343-s001.zip › Supplementary File/All Supplementary Tables.doc]

**Table S1 Primers used for qPCR**

| Genes | Primer sequences | |
| --- | --- | --- |
| *ACTIN* | Up: CACTCTCCTTCAGCATCTCA | Down: CATCAATATCGAGATATGCCTCCTTCA |
| *CAO1* | Up: ACTCAACAGATGGGAAATGC | Down: ACGGAGGAAGTAAACAAGGA |
| *PDS1* | Up: CGGGTATTATGAGAGGGATG | Down: GAAATGGACAGTGGTTGGAT |
| *PSY3* | Up: GGTATGGGCTTCATTGCTGT | Down: CGGGCGTAAGAGGGATTT |
| *CHLM* | Up: GCTTCGGCGTGGAGATAAG | Down: GATGGGATGATTGCTCACCT |
| *CCS* | Up: TTTAGTCCATCCTTCAACTG | Down: CAACCCATTCCACACTTTCT |
| *NCED1* | Up: CACCGAAATCACTTGCTCT | Down: TGGGTTGCTGCTTAGTTGTA |
| *CLH* | Up: AACGGAGAAGTTAACGATGC | Down: CCAGACCGAACGCTATTAGT |
| *PAO* | Up:GTTTCCTCAGTATTCCCTTTG | Down: TGTGGTTGCTTGTTCCTAAA |
| *GLuRS* | Up: TATTGAGACATTGGGCATCA | Down: ATTCTATCCCGTCCATCCTT |
| *HEMG* | Up: GTTGTTGGTGCTGGTGTTAG | Down: ATTGTGTTTGCTCCCTCATC |
| *ALAD1* | Up: TTTCCAGCCTTGTCCATACT | Down: ACGAGACCTTGGGTTCAATA |
| *FC1* | Up: GCCCGTAAATGTCTATGTTGG | Down: TTGTGGATAAAGTGGCAGCA |
| *AB1* | Up: CCGATTATGTTGTCTGCGATT | Down: GGCAAGAAAGAGGCTAAATGA |
| *PSY7* | Up: CGACATTCAGCCATTCAGAG | Down: CCTACGGTCCCAGCAACATA |
| *PSY5* | Up: CGACATTCAGCCATTCAGAG | Down: GCCCATAACTGGAACACTCA |
| *PSY6* | Up: TATGGGCTTCATTGCTGTTG | Down: CGGGCGTAAGAGGGATTT |
| *HEMF1* | Up: CGCTTCCTCTTCGTTCACTC | Down: TTGTCGTCGTGGTCATCTTG |

**Table S2** Primers used for gene cloning

| Genes | Primer sequences | |
| --- | --- | --- |
| *CHLM* | Up: ATGGCATTCACAGCTTCACTGTCTTC | Down: CTATGCAGGGACAGCCTCAACAAG |
| *DVR* | Up: ATGGCCCTCCAAGCTGCTGCTT | Down: TTAGGCCAAACCAACAAGTTTCTCAC |
| *CLH* | Up: ATGACAGCATTAGCCGATAGCAAG | Down: TTAAGCTTTTGCACGAGCTGCG |
| *CCS* | Up: GCTCTGGCATGGCAACTCTTCTTAG | Down: CTTCAAATGGTTTCAAGGGCAAGATTC |
| *PSY3* | Up: CAGCATGTCTGTTACATTGCTGT | Down: GTTCAGTTTAAGCCTTACTGGT |
| *PSY5* | Up: CAGCATGTCTGTTACATTGCTGT | Down: GGCTTTACTCACATAAGCTCTCT |
| *PSY6* | Up: CAGCATGTCTGTTACATTGCTGTG | Down: GTTCAGTTTAAGCCTTACTGGTAT |
| *PSY7* | Up: CAGCATGTCTGTTACATTGCTGT | Down: GTGTACTTACTCCTCTCCAACATC |
| *NCED1* | Up: ATGGCGGCAGCAACTACTACTTCT | Down: TTAGGCCTGCTTGGCCAAATCTT |
| *AB* | Up: ATGGATTTTACTTTCTTGTTTCTC | Down: TTACATGTTGTGAATTGTGATTTCG |

**Table S3** Statistics of annotation of transcripts

| Databases | Total number | COG | GO | KEGG | Swiss-Port | Nr |
| --- | --- | --- | --- | --- | --- | --- |
| Gene No. | 299 | 55 | 222 | 39 | 189 | 297 |

**Table S4** Statistics of annotation of differentially expressed transcripts

| Comparison | nr | SwissPort | GO | KEGG | COG |
| --- | --- | --- | --- | --- | --- |
| T2 vs T1 | 2,954 | 2,400 | 2,648 | 485 | 1,288 |
| T1 vs T3 | 2,647 | 2,111 | 2,363 | 413 | 1,075 |
| T2 vs T3 | 1,806 | 1,457 | 1,640 | 319 | 784 |
