# Supplementary material for: Comparative transcriptome analyses of a late-maturing mandarin mutant and its original cultivar reveals gene expression profiling associated with citrus fruit maturation
Source: PeerJ. 2017 May 18;5:e3343. doi: 10.7717/peerj.3343 (PMC5438589; doi:10.7717/peerj.3343)
Supplement: File S1 [file peerj-05-3343-s001.zip › Supplementary File/Table S1.doc]

| Genes | Primer sequences | |
| --- | --- | --- |
| *ACTIN* | Up: CACTCTCCTTCAGCATCTCA | Down: CATCAATATCGAGATATGCCTCCTTCA |
| *CAO1* | Up: ACTCAACAGATGGGAAATGC | Down: ACGGAGGAAGTAAACAAGGA |
| *PDS1* | Up: CGGGTATTATGAGAGGGATG | Down: GAAATGGACAGTGGTTGGAT |
| *PSY3* | Up: GGTATGGGCTTCATTGCTGT | Down: CGGGCGTAAGAGGGATTT |
| *CHLM* | Up: GCTTCGGCGTGGAGATAAG | Down: GATGGGATGATTGCTCACCT |
| *CCS* | Up: TTTAGTCCATCCTTCAACTG | Down: CAACCCATTCCACACTTTCT |
| *NCED1* | Up: CACCGAAATCACTTGCTCT | Down: TGGGTTGCTGCTTAGTTGTA |
| *CLH* | Up: AACGGAGAAGTTAACGATGC | Down: CCAGACCGAACGCTATTAGT |
| *PAO* | Up:GTTTCCTCAGTATTCCCTTTG | Down: TGTGGTTGCTTGTTCCTAAA |
| *GLuRS* | Up: TATTGAGACATTGGGCATCA | Down: ATTCTATCCCGTCCATCCTT |
| *HEMG* | Up: GTTGTTGGTGCTGGTGTTAG | Down: ATTGTGTTTGCTCCCTCATC |
| *ALAD1* | Up: TTTCCAGCCTTGTCCATACT | Down: ACGAGACCTTGGGTTCAATA |
| *FC1* | Up: GCCCGTAAATGTCTATGTTGG | Down: TTGTGGATAAAGTGGCAGCA |
| *AB1* | Up: CCGATTATGTTGTCTGCGATT | Down: GGCAAGAAAGAGGCTAAATGA |
| *PSY7* | Up: CGACATTCAGCCATTCAGAG | Down: CCTACGGTCCCAGCAACATA |
| *PSY5* | Up: CGACATTCAGCCATTCAGAG | Down: GCCCATAACTGGAACACTCA |
| *PSY6* | Up: TATGGGCTTCATTGCTGTTG | Down: CGGGCGTAAGAGGGATTT |
| *HEMF1* | Up: CGCTTCCTCTTCGTTCACTC | Down: TTGTCGTCGTGGTCATCTTG |
