# Supplementary material for: Comparative transcriptome analyses of a late-maturing mandarin mutant and its original cultivar reveals gene expression profiling associated with citrus fruit maturation
Source: PeerJ. 2017 May 18;5:e3343. doi: 10.7717/peerj.3343 (PMC5438589; doi:10.7717/peerj.3343)
Supplement: File S1 [file peerj-05-3343-s001.zip › Supplementary File/Table S2.doc]

| Genes | Primer sequences | |
| --- | --- | --- |
| *CHLM* | Up: ATGGCATTCACAGCTTCACTGTCTTC | Down: CTATGCAGGGACAGCCTCAACAAG |
| *DVR* | Up: ATGGCCCTCCAAGCTGCTGCTT | Down: TTAGGCCAAACCAACAAGTTTCTCAC |
| *CLH* | Up: ATGACAGCATTAGCCGATAGCAAG | Down: TTAAGCTTTTGCACGAGCTGCG |
| *CCS* | Up: GCTCTGGCATGGCAACTCTTCTTAG | Down: CTTCAAATGGTTTCAAGGGCAAGATTC |
| *PSY3* | Up: CAGCATGTCTGTTACATTGCTGT | Down: GTTCAGTTTAAGCCTTACTGGT |
| *PSY5* | Up: CAGCATGTCTGTTACATTGCTGT | Down: GGCTTTACTCACATAAGCTCTCT |
| *PSY6* | Up: CAGCATGTCTGTTACATTGCTGTG | Down: GTTCAGTTTAAGCCTTACTGGTAT |
| *PSY7* | Up: CAGCATGTCTGTTACATTGCTGT | Down: GTGTACTTACTCCTCTCCAACATC |
| *NCED1* | Up: ATGGCGGCAGCAACTACTACTTCT | Down: TTAGGCCTGCTTGGCCAAATCTT |
| *AB* | Up: ATGGATTTTACTTTCTTGTTTCTC | Down: TTACATGTTGTGAATTGTGATTTCG |
