# Supplementary material for: Comparative transcriptome analyses of a late-maturing mandarin mutant and its original cultivar reveals gene expression profiling associated with citrus fruit maturation
Source: PeerJ. 2017 May 18;5:e3343. doi: 10.7717/peerj.3343 (PMC5438589; doi:10.7717/peerj.3343)
Supplement: File S1 [file peerj-05-3343-s001.zip › Supplementary File/Table S3.doc]

| Databases | Total number | COG | GO | KEGG | Swiss-Port | Nr |
| --- | --- | --- | --- | --- | --- | --- |
| Gene No. | 299 | 55 | 222 | 39 | 189 | 297 |
