# Supplementary material for: Comparative transcriptome analyses of a late-maturing mandarin mutant and its original cultivar reveals gene expression profiling associated with citrus fruit maturation
Source: PeerJ. 2017 May 18;5:e3343. doi: 10.7717/peerj.3343 (PMC5438589; doi:10.7717/peerj.3343)
Supplement: File S1 [file peerj-05-3343-s001.zip › Supplementary File/Table S4.doc]

| Comparison | nr | SwissPort | GO | KEGG | COG |
| --- | --- | --- | --- | --- | --- |
| T2 vs T1 | 2,954 | 2,400 | 2,648 | 485 | 1,288 |
| T1 vs T3 | 2,647 | 2,111 | 2,363 | 413 | 1,075 |
| T2 vs T3 | 1,806 | 1,457 | 1,640 | 319 | 784 |
